# Pachychoroid neovasculopathy versus macular neovascularization in age-related macular degeneration with and without shallow irregular pigment epithelial detachment

**DOI:** 10.1038/s41598-023-46891-6

**Published:** 2023-11-09

**Authors:** Hamid Riazi-Esfahani, Esmaeil Asadi Khameneh, Fariba Ghassemi, Mohammadreza Mehrabi Bahar, Ali torkashvand, Alireza Mahmoudi, Ahmed Husein Ahmed, Shahin Faghihi, Masoud Rahimi, Ali Akbarzadeh, Hooshang Faghihi, Elias Khalili Pour

**Affiliations:** 1 Farabi Eye Hospital, Tehran University of Medical Sciences, Tehran, Iran; 2 Noor Eye Institute, Tehran, Iran; 3 Doheny Eye Institute, Pasadena, CA USA; 4 Eye Research Center, Farabi Eye Hospital, Qazvin Sq, Tehran, Iran

**Keywords:** Diagnostic markers, Macular degeneration, Retinal diseases, Vision disorders

## Abstract

To compare the choroidal neovascular features of individuals with pachychoroid neovasculopathy (PNV) and neovascular age-related macular degeneration (nAMD) with and without shallow irregular pigment epithelial detachment (SIPED). Using optical coherence tomography angiography, the choroidal neovascular complexes of 27 patients with PNV, 34 patients with nAMD and SIPED, and 15 patients with nAMD without SIPED were analyzed with FIJI and AngioTool software. PNV compared to nAMD with SIPED had a greater vessel percentage area (P = 0.034), junction density (P = 0.045), average vessel length (P < 0.001), and fractal dimension (P < 0.001). PNV, compared to nAMD without SIPED, had a greater total vessel length (P = 0.002), total number of junctions (P < 0.001), junction density (P = 0.034), and fractal dimension (P = 0.005). nAMD with SIPED, compared to nAMD without SIPED, had greater vessel area, total number of junctions, total vessel length, and average vessel length (all P values < 0.001). Patients with nAMD plus SIPED and individuals with nAMD without SIPED have similar fractal dimension values (P = 0.703). Biomarkers of choroidal neovascular complexity, such as fractal dimension, can be used to differentiate PNV from nAMD with or without SIPED.

## Introduction

Age-related macular degeneration (AMD) is one of the leading causes of blindness in the elderly^1^. The most common methods used to identify macular neovascularization (MNV) due to neovascular age-related macular degeneration (nAMD) include fundoscopy and imaging techniques, such as optical coherence tomography (OCT), fluorescein angiography (FA), indocyanine green angiography (ICGA), and optical coherence tomography angiography (OCTA). Pachychoroid neovasculopathy (PNV) is characterized by the presence of type 1 choroidal neovascularization (CNV), often inside a shallow irregular pigment epithelial detachment (SIPED) in the setting of pachychoroid spectrum disorders characterized by increased choroidal thickness (pachychoroid), increased outer choroidal vascular caliber (pachyvessels), and choriocapillaris thinning on enhanced depth OCT (EDI-OCT)^2,3^. A growing body of research indicates that pachychoroid-driven MNV and AMD-driven MNV are distinct entities. They differ in terms of demographics, pathogenesis, treatment response, and prognosis^2,4^. Further genetic investigations have demonstrated that pachychoroid spectrum disorders have a distinct genetic profile from the drusen-driven MNV^5–7^. Consequently, it is essential to distinguish between these two groups. On the other hand, a number of studies have indicated that pachychoroid-driven CNV is frequently misdiagnosed as nAMD^6,8^.

With the advent of OCTA, assessments of neovascular membranes are becoming more sophisticated. OCTA is a non-invasive imaging technique that allows healthcare professionals to obtain a depth-resolved view of the macular neovascular membranes, as well as the extent and complexity of these structures^9^. Different studies have been performed to investigate the anatomical and pathological choriocapillaris changes in non-neovascular and neovascular age-related macular degeneration as well as other choroidal vascular disorders^10–14^. OCTA biomarkers such as the number of junction points, fractal dimension, and lacunarity have shown potential in assessing the complexity of neovascular membranes^15–17^. These biomarkers have been studied in previous investigations and have demonstrated the abilities to provide valuable information about the nature of these abnormal blood vessels and to predict treatment responses and disease severity^15,18,19^.

While SIPED is an OCT marker for PNV, it has also been observed in individuals with various disorders in the pachychoroid spectrum disease and in those with nAMD^2,4^.

In this study, we investigated the complexity of neovascular membranes in three patient groups: PNV patients with SIPED as a structural OCT feature, nAMD patients with and without SIPED, to evaluate if this OCT marker gives different neovascular network characteristics in nAMD patients versus PNV patients.

## Results

A total of 123 eyes of 120 patients were included in this study. After excluding low-quality OCTA images and MNV complexes that were not completely within the 3 × 3 mm image area, 76 eyes remained for further analysis. Of the 76 eyes that were analyzed, 27 had PNV and SIPED (Group 1), 34 had nAMD and SIPED (Group 2), and 15 had nAMD without SIPED (Group 3). The mean age of the patients was 58.30 ± 10.52 years in Group 1 (range 45–74 years), 68.21 ± 6.01 years in Group 2 (range 55–80 years), and 69.00 ± 5.14 years in Group 3(range 63–76 years). The patients in Group 1 were significantly younger than those in Groups 2 and 3, while the difference between Groups 2 and 3 was not statistically significant (P = 0.008, P = 0.005, and P = 0.951, respectively). The rates of right eye involvement were 40.7%, 52.9%, and 46.7% in Groups 1, 2, and 3, respectively, and there were no statistically significant differences between the groups (P = 0.637). There was a statistically significant male predominance in Groups 2 (64.7%) and 3 (86.7%) in comparison to Group 1 (44.4%) (P = 0.024). The mean LogMAR best corrected visual acuity (BCVA) values for Groups 1, 2, and 3 were 0.71 ± 0.35, 0.61 ± 0.31, and 0.81 ± 0.42, respectively, which do not present any statistically significant differences between these three groups (P = 0.196).

**Figure 1:**
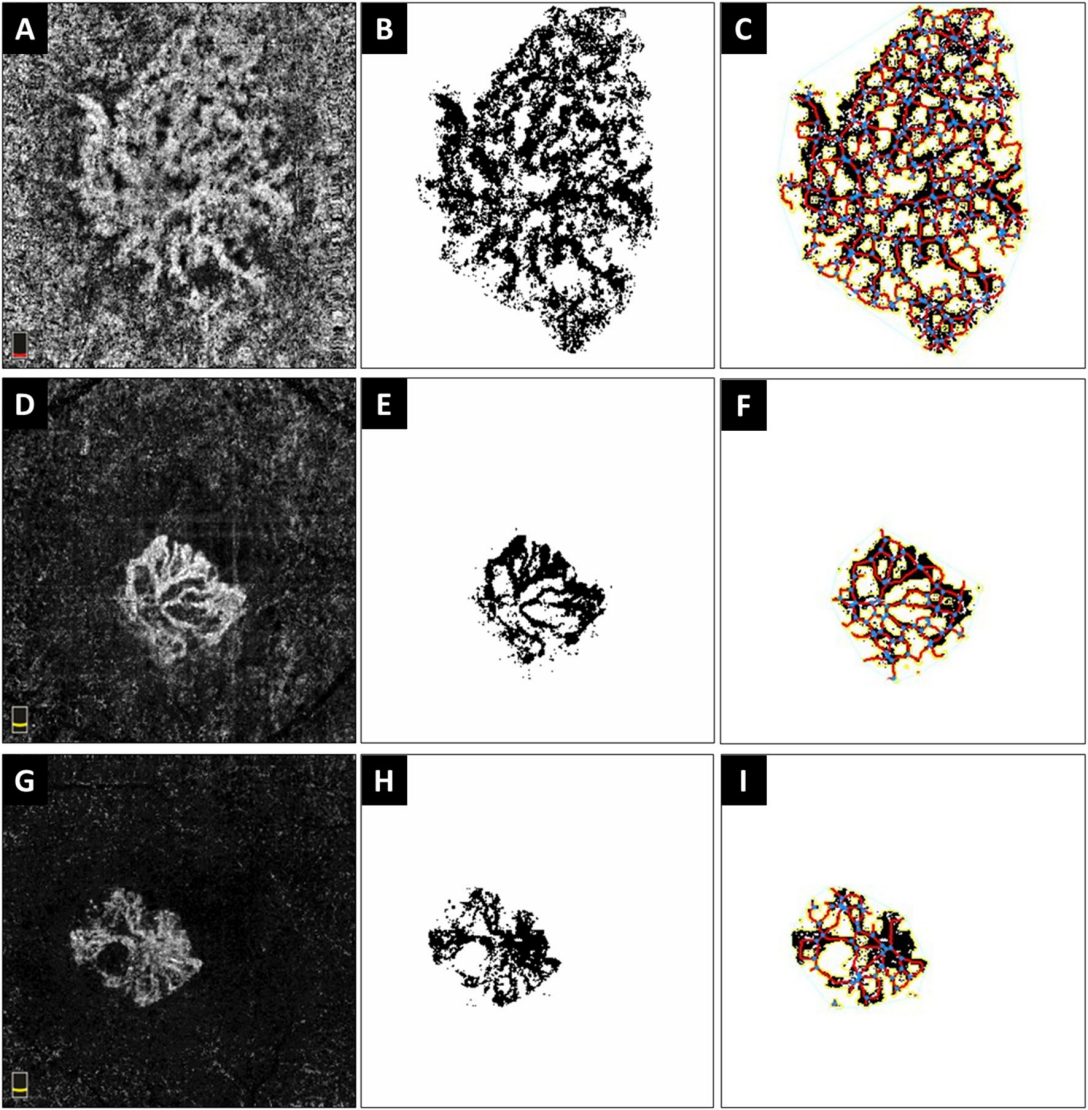
Optical coherence tomography angiography analysis of choroidal neovascular complex of a patient with pachychoroid neovasculopathy (PNV) with shallow irregular pigment epithelial detachment (SIPED) (**A**–**C**), patient with neovascular age-related macular degeneration (nAMD) with SIPED (**D**–**F**), and patient with nAMD without SIPED (**G**–**I**). The first column demonstrates choroidal neovascular complex in choriocapillaris (**A**) and outer retina slabs (**D** and **G**), the second column shows binarized neovascular complexes which were extracted from the rest of the image, and the third column shows the results of analysis that was performed by AngioTool. In the first patient (**A**–**C**), the vessel percentage area was 63.89%, fractal dimension was 1.656, and mean lacunarity was 0.114. In the second patient (**D**–**F**), the vessel percentage area was 56.01%, fractal dimension was 1.522, and mean lacunarity was 0.158. In the third patient (**G**–**I**), the vessel percentage area was 61.29%, fractal dimension was 1.539, and lacunarity was 0.125.

Analysis of the MNV complex quantitative characteristics is summarized in Table 1. The mean junction density was significantly higher in Group 1 in comparison to those in both Group 2 (P = 0.045) and Group 3 (P = 0.034). However, the difference between Groups 2 and 3 was not statistically significant (P = 0.884). The mean fractal dimensions were 1.598 ± 0.017, 1.514 ± 0.015, and 1.506 ± 0.026 in Groups 1, 2, and 3, respectively. The value was significantly higher in Group 1 in comparison to Groups 2 (P < 0.001) and 3 (P = 0.005), while the difference between Groups 2 and 3 was not statistically significant (P = 0.703). Figure 1 represents some of the MVN characteristics of a patient with PNV, nAMD with SIPED, and nAMD without SIPED.

**Table 1:** Choroidal neovascular complex characteristics of patients.

Variable	Study groups—Mean ± SD			Adjusted P values†			
	PNV (Group 1)	nAMD with SIPED (Group 2)	nAMD without SIPED (Group 3)	Overall	Groups 1 vs 2	Groups 1 vs 3	Groups 2 vs 3
Explant area (mm^2^)	2.69 ± 0.32	1.88 ± 0.23	1.36 ± 0.31	< 0.001*	0.995	0.002*	< 0.001*
Vessel area (mm^2^)	1.69 ± 0.21	1.01 ± 0.13	0.78 ± 0.19	< 0.001*	0.811	< 0.001*	< 0.001*
Vessel percentage area	62.56 ± 1.55	55.48 ± 1.59	59.02 ± 2.78	0.035*	0.034*	0.980	0.594
Total number of junctions	156.26 ± 20.96	96.12 ± 14.98	69.60 ± 18.36	< 0.001*	0.972	< 0.001*	< 0.001*
Junction density (n/mm)	55.44 ± 1.02	49.28 ± 1.02	47.22 ± 2.05	0.024*	0.045*	0.034*	0.884
Total vessel length (mm)	26.46 ± 4.24	16.93 ± 2.36	12.64 ± 3.06	< 0.001*	0.995	0.002*	< 0.001*
Average vessel length (mm)	16.78 ± 3.04	7.69 ± 1.65	4.08 ± 0.87	< 0.001*	< 0.001*	0.602	< 0.001*
Total number of end points	49.73 ± 1.16	31.99 ± 1.10	21.53 ± 3.42	0.169	0.972	0.285	0.213
Lacunarity	0.117 ± 0.011	0.162 ± 0.013	0.136 ± 0.018	0.056	0.065	0.798	0.557
Dispersion	554.39 ± 528.27	64.94 ± 48.79	1021.20 ± 970.63	0.518	0.591	0.641	0.679
Fractal dimension	1.598 ± 0.017	1.514 ± 0.015	1.506 ± 0.026	< 0.001*	< 0.001*	0.005*	0.703

Statistically significant P values are indicated by an asterisk (*).

PNV, pachychoroid neovasculopathy; nAMD, neovascular age-related macular degeneration; SIPED, shallow irregular pigment epithelial detachment.

^†^The reported P values are adjusted for age and sex.

The mean vessel area, total number of junctions, and total vessel length were significantly higher in Group 1 in comparison to Group 3 (P < 0.001, < 0.001, and 0.002, respectively) and in Group 2 in comparison to Group 3 (P < 0.001, < 0.001, and < 0.001, respectively); the differences between Groups 1 and 2 (P = 0.811, 0.972, and 0.995, respectively) was not statistically significant.

The mean vessel percentage area was 62.56 ± 1.55, 55.48 ± 1.59, and 59.02 ± 2.78 in Groups 1, 2, and 3, respectively, which was significantly higher in Group 1 than in Group 2 (P = 0.034). However, the difference between Groups 1 and 3 (P = 0.980) and Groups 2 and 3 (P = 0.594) was not statistically significant.

The mean average vessel length was significantly higher in Group 1 in comparison to Group 2 (P < 0.001) and in Group 2 in comparison to Group 3 (P < 0.001); the differences between Groups 1 and 3 (P = 0.602) was not statistically significant.

**Figure 2:**
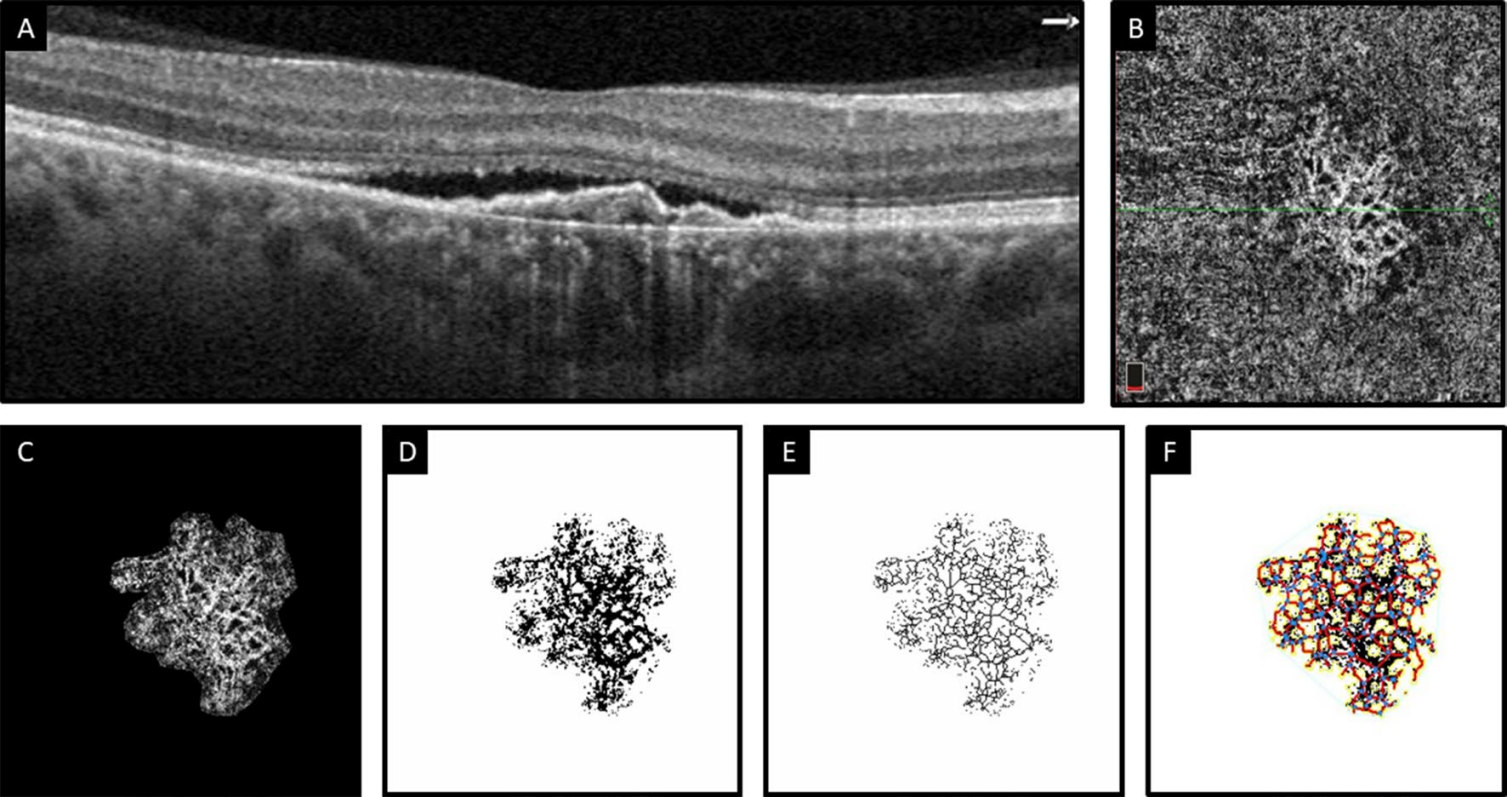
Multimodal imaging of a patient with pachychoroid neovasculopathy. (**A**) Optical coherence tomography angiography B-scan image demonstrates subretinal fluid, shallow irregular pigment epithelial detachment, and pachyvessels. (**B**) En face choriocapillaris slab of optical coherence tomography angiography shows choroidal neovascular complex. The horizontal green line indicates the position of the corresponding B-scan. (**C**) The neovascular complex that is extracted from the rest of the choriocapillaris slab after adjusting the mean threshold using ImageJ. (**D** and **E**) Corresponding binarized and skeletonized images of choroidal neovascular complex. (**F**) Choroidal neovascular complex processed by AngioTool showing the border of vascular complex (yellow lines) and vascular junctions (blue dots) based on skeletonized vessels (red lines).

**Figure 3:**
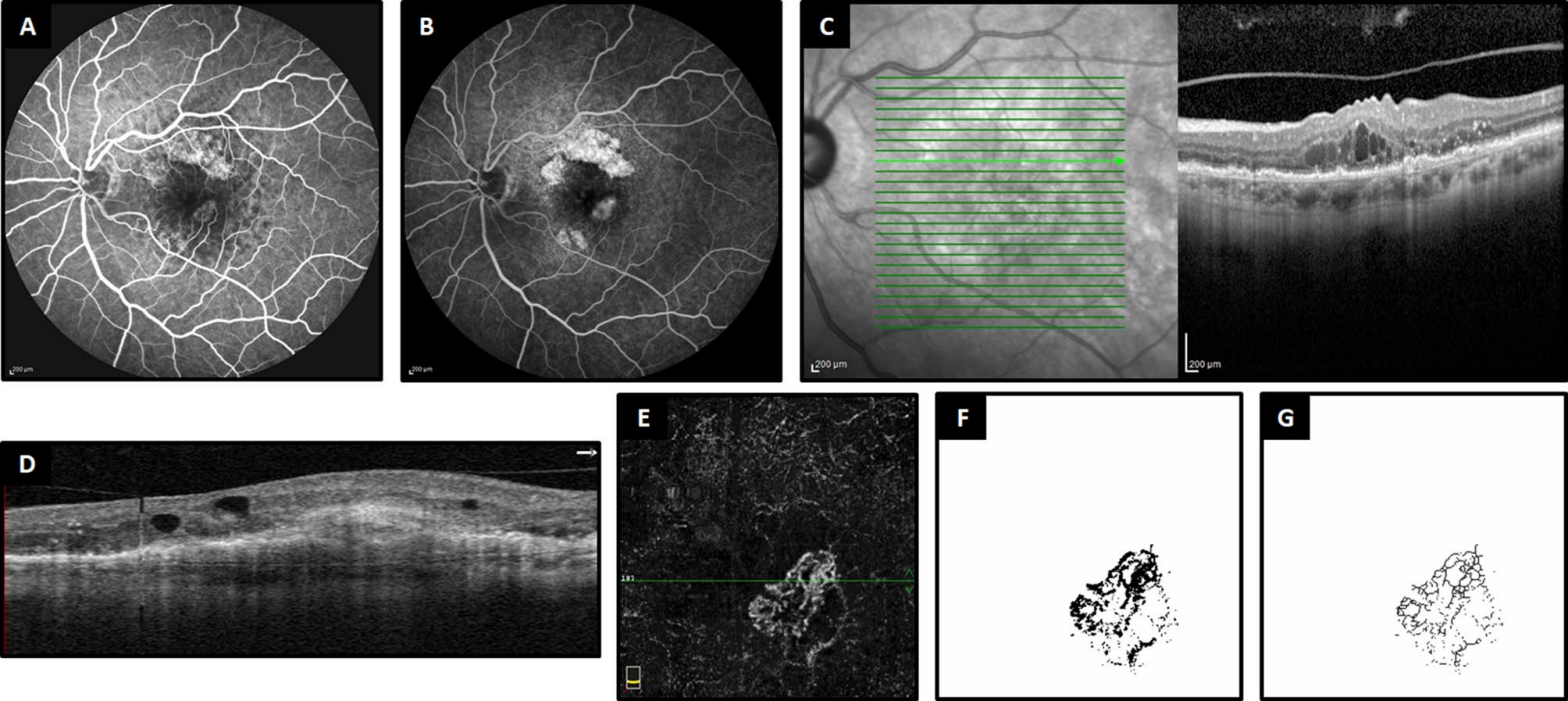
Multimodal imaging of a patient with neovascular age-related macular degeneration. In (**A**) and (**B**), fluorescein angiography reveals an early hyperfluorescent lesion in the macular area (**A**) and late leakage indicative of active choroidal neovascularization (**B**). In (**C**), the green arrow on the infrared image points to a corresponding B-scan from optical coherence tomography, displaying intraretinal fluid with shallow irregular pigment epithelial detachment. (**D**) Optical coherence tomography angiography B-scan also shows intraretinal fluid. In (**E**), the en face outer retina slab of optical coherence tomography angiography highlights the choroidal neovascular complex, with the horizontal green line indicating the position of the corresponding B-scan. In (**F**) and (**G**), the corresponding binarized and skeletonized images of the choroidal neovascular complex are presented.

**Figure 4:**
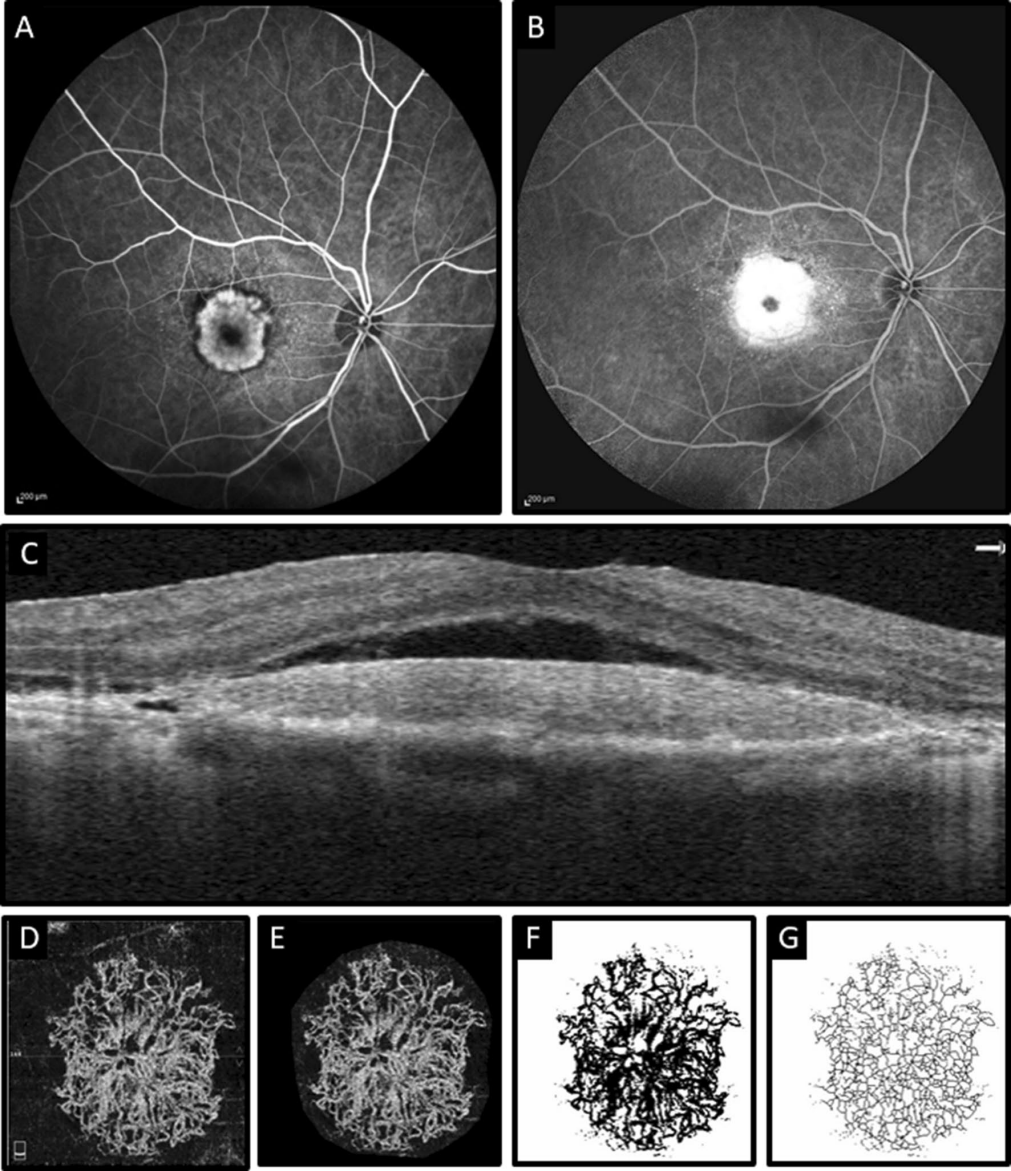
Multimodal imaging of a patient with neovascular age-related macular degeneration. (**A**) and (**B**): Fluorescein angiography indicates early hyperfluorescent lesion (**A**) in macular area with late leakage (**B**). (**C**) Optical coherence tomography angiography B-scan shows subretinal fluid with subretinal hyperreflective material without shallow irregular pigment epithelial detachment. (**D**) En face outer retina slab of optical coherence tomography angiography shows choroidal neovascular complex. The horizontal green line indicates the position of the B-scan. (**E**) The neovascular complex that is extracted from the rest of the outer retina slab after adjusting the mean threshold with ImageJ. (**F**) and (**G**) corresponding binarized and skeletonized images of choroidal neovascular complex.

The mean lacunarity values were 0.117 ± 0.011, 0.162 ± 0.013, and 0.136 ± 0.018 in Groups 1, 2, and 3, respectively. The differences between Groups 1, 2, and 3 was not statistically significant (P = 0.056). The mean dispersion values were 554.39 ± 528.27, 64.94 ± 48.79, and 1021.20 ± 970.63 in Groups 1, 2, and 3, respectively. None of the differences between groups were statistically significant (P = 0.518). The mean total numbers of end points were 49.73 ± 1.16 in Group 1, 31.99 ± 1.10 in Group 2, and 21.53 ± 3.42 in Group 3. There were no statistically significant differences between all three groups (P = 0.169). Figures 2, 3, and 4 represent the multimodal imaging of patients with PNV, nAMD with SIPED, and nAMD without SIPED, respectively, along with the corresponding binarized and skeletonized images.

## Discussion

Pang and Freund described choroidal neovascularization in three cases that did not have the typical changes of nAMD and were not driven by myopia. Instead, they found that the characteristics of the eyes were more consistent with pachychoroid spectrum diseases. They also introduced the term “PNV” into the literature and speculated that this type of choroidal neovascularization is distinct from those of typical AMD-related CNVs^6^. Although nAMD has been described as a separate clinical entity from PNV, it is still under debate whether PNV is a variant of nAMD. Different studies have shown that about 15–20% of patients with PNV were misdiagnosed as having nAMD, and there is a considerable overlap between these two entities^8,20^.

Originally, the term SIPED was described in chronic central serous chorioretinopathy (CSC) cases, and later, several studies detected neovascular tissue beneath the lesions^21^. Bousquet et al.^22^ found that OCTA of SIPED in patients with chronic CSC reveals neovascularization in 35% of cases, while a combination of OCTA, FA, and ICGA was representative for MNV in 25% of cases. This finding signifies the role of OCTA in detecting the vascular complex under SIPEDs. However, the presence of MNV in SIPED has been reported at rates ranging from 24 to 95%^22–27^; this discrepancy could be related to the absence of well-defined criteria.

To our knowledge, this is the first study to compare the quantitative vascular features of MNV complex in patients with PNV and nAMD based on the presence or absence of SIPED.

In the current study, patients with PNV were significantly younger than patients with nAMD with or without SIPED. The baseline best corrected visual acuity was comparable between three groups. Compared to patients with nAMD and SIPED, patients with PNV had a greater vessel percentage area, junction density, average vessel length, and fractal dimension. Patients with PNV had a greater vessel area, total number of junctions, junction density, total vessel length, and fractal dimension than patients with nAMD without SIPED. Patients with nAMD with SIPED compared to patients with nAMD without SIPED had greater vessel area, total number of junctions, total vessel length, and average vessel length.

The finding that patients with PNV are younger than those with nAMD was replicated in the present investigation^3,28^. Although previous studies^3,28,29^ indicated that patients with PNV had better visual acuity than those with nAMD due to a lower rate of macular scar formation, our study demonstrates that the baseline visual acuity of patients with PNV and nAMD is comparable. However, the visual outcomes of these two entities after treatment were not included in this report.

Fractal dimension, dispersion, and lacunarity have been studied as quantitative measures of neovascular network characteristics. The utilization of quantitative optical coherence tomography angiography analysis has the potential to provide an objective means of quantifying aberrations in vascular complexes. Various vascular complexity characteristics exist, such as the vessel complexity index, fractal dimension, lacunarity, vascular dispersion, and tortuosity. Each of these criteria quantified the complexity by considering distinct components of a vascular network and used mathematical methods. The concept of fractal dimension serves as a quantitative measure of the detailed complexity of a fractal pattern, which exhibits variations when the scale of observation is altered^30^. The fractal dimension is an index of morphological complexity ranging from 0 to 2; the higher the index, the greater the complexity. Patients with PNV had numerically greater fractal dimension than both groups of nAMD, and this difference was statistically significant. We interpret that the greater fractal dimension index in patients with PNV was attributed to more complex MNV in these patients in comparison to patients with nAMD. Particularly, FD, which provides insights into the architecture and complexity of a vascular network, is a useful biomarker for distinguishing the remission of Type 1 MNV from treatment-naive quiescent MNV, two categories of MNV with distinct natural histories and prognoses^16^. Similarly, a significant difference in FD has been observed between Type 1 MNVs in active versus remission states^16^. Overall, FD appears to be a promising biomarker for assessing the structure of MNVs, as it may shed light on the underlying pathogenesis and aid in treatment planning. In future applications, mathematical methodologies and complexity metrics are anticipated to be employed in imaging machine plugins. Additional research is required to thoroughly comprehend the relationship between FD and MNV in various subtypes of AMD (with or without SIPED) and PNV.

Dispersion quantifies the disorganization of the vascular network. The greater the value, the more disorganized the vascular network. Another quantitative metric that reflects structural non-uniformity or non-homogeneity is lacunarity. Serra et al., reported that vascular perfusion density was substantially reduced in patients with PCV compared to those with nAMD. They also found that fractal dimension was not significantly different between PCV and nAMD patients and that lacunarity was greater in patients with PCV^1^. Although this study compares two distinct groups of patients previously treated with anti-vascular endothelial growth factor (Anti-VEGF) agents, our study on treatment-naive patients demonstrates that the percentage area of vessels was significantly greater in patients with PNV versus nAMD with SIPED. In our study, the difference in FD between the PNV and nAMD groups indicates a variation in neovascular organization and branching complexity. Our study’s strength lies in the fact that we included treatment-naive eyes. Recent OCTA studies have demonstrated that neovascular lesions may experience microvascular alterations as a result of treatment with anti-VEGFs. Specifically, anti-VEGFs have been hypothesized to facilitate vascular remodeling^31,32^.

Arf et al.^29^ compared MNV complex in patients with nAMD and PNV and found no significant differences between lesion size and flow area. However, in the current study, we showed that patients with PNV had a substantially larger vessel area than patients with nAMD without SIPED; moreover, the explant area of MNV complex in patients with PNV was significantly greater than in patients with nAMD without SIPED.

Altinisik et al.^33^ found that morphological aspects of the neovascular complex may have a minor role in distinguishing PNV from nAMD. Most morphological assessments in their study were based on qualitative indices, but we were able to show that there is at least a statistically significant difference between the neovascular networks of patients with PNV and nAMD by considering several quantitative vascular complex characteristics.

Although we have demonstrated in a previous study on patients with CSC that choroidal biomarkers, such as choroidal thickness and choroidal vascularity index, can influence the presence or absence of MNV under SIPED, it remains to be determined whether the choroid can also influence the MNV characteristics of patients with PNV and nAMD with or without SIPED^34^. Our study has no enough power to answer this question and larger studies are required to answer this question. However, it appears that there are morphological differences in the characteristics of the MNVs of these three groups, and these differences may be influenced by their anatomical or structural differences and the characteristics of their choroids.

In this study, we found that patients with nAMD with SIPED compared to patients with nAMD without SIPED had different neovascular network characteristics as the vessel area, total number of junctions, total vessel length, and average vessel length were significantly greater in patients with nAMD and SIPED.

This study was limited by its retrospective nature and the small size of each subgroup. Furthermore, we only recruited cases with sufficient OCTA quality for quantitative analysis; a substantial number of cases were excluded due to significant artifacts or low-quality images, which are among the most common limitations of OCTA. Another limitation of our study is that we did not use a swept-source OCTA for the detection of choroidal neovascularization in our patients. In addition, we did not analyze patients with either type 1 or mixed type macular neovascularization separately, due to the cross-sectional nature of this investigation, we were also unable to assess longitudinal changes in the vascular networks in response to various interventions. This investigation was conducted at a tertiary care facility, which may have resulted in the recruitment of a disproportionate number of chronic cases and selection bias. Finally, the duration of symptoms that could impact the morphological characteristics of the neovascular membrane was not specified.

In conclusion, our study demonstrates that patients with PNV have more complex morphological MNV characteristics than patients with nAMD, regardless of the presence of SIPED. This distinction may contribute to variable treatment responses for these two entities. Additional research with larger sample sizes and which evaluates these OCTA biomarkers is needed.

## Methods

This study was a retrospective case series analysis of patients diagnosed with nAMD or PNV at the retina clinic of the Farabi Eye Hospital (Tehran, Iran) between March 2019 and September 2022. Informed consent was obtained from all subjects or their legal guardians. The study was approved by the institutional review board committee of Tehran University of Medical Sciences (Ethical code: IR.TUMS.FARABIH.REC.1401.008) and adhered to the Declaration of Helsinki.

Inclusion criteria for nAMD groups were treatment-nave patients aged more than 50 years with nAMD confirmed by two retina specialists (H.R. and E.K.) based on fundus examination, macular OCT (Spectralis SDOCT, Heidelberg Engineering, Germany) associated with drusen, subretinal and/or intraretinal fluid, and leakage on FA (Spectralis HRA + OCT; Heidelberg Engineering, Heidelberg, Germany). Patients with either type 1 or mixed type macular neovascularization were included. Based on a clinical exam, OCT, OCTA, and FA, pachychoroid spectrum disorders and other causes of CNV were ruled out. When the diagnosis was ambiguous, EDI-OCT and ICGA were performed.

Patients with pachychoroid detected by EDI-OCT, choroidal vascular hyperpermeability on ICGA, choroidal neovascularization associated with SIPED and SRF, and/or IRF on OCTA met the inclusion criteria for the PNV group^34^.

Patients with diabetes mellitus, refractive error (spherical equivalent) ≥  ± 4 diopters, other ocular disorders, a history of ocular surgery (with the exception of cataract surgery), systemic hypertension (systolic BP > 150 mm Hg or diastolic BP > 90 mm Hg despite receiving medical treatment), and media opacity were excluded. Those who were found to have polypoidal choroidal vasculopathy (PCV) according to an ICGA diagnosis were also excluded.

Two retina specialists (H.R. and E.K.) evaluated all cross-sectional raster B-scans from each patient’s macular OCT to detect SIPED, which was defined as a hyperreflective Bruch’s membrane band beneath irregularly elevated retinal pigmented epithelium (RPE)^22,35^. A third retina specialist (E.A.) examined the OCT scans if the diagnosis was ambiguous. Patients were divided into three categories based on the presence or absence of SIPED. Patients with PNV and SIPED comprised Group 1, patients with nAMD and SIPED comprised Group 2, and nAMD patients without SIPED comprised Group 3.

All patients underwent macular OCTA (RTVue-XR; Optovue, Inc., Freemont, CA) with 3 × 3 mm and 6 × 6 mm cube size scans centered at the level of the macula. Projection artifact elimination was performed using the device’s in-built software. The retina en face slabs are defined as the outer retina (between the outer boundary of the outer plexiform layer to Bruch’s membrane) and choriocapillaris (from the Bruch’s membrane line to 30 μm below the line). On the outer retina and choriocapillaris en face images generated by automatic segmentation obtained from the OCTA’s built-in software, the presence of neovascular complex was evaluated; if necessary, segmentations were manually corrected^36^. To better visualize the MNV complex, the en face image boundaries were adjusted manually to encompass the entire lesion. Low-quality images (< 6/10) and those with MNV complexes that were partially or entirely outside the 3 × 3 mm cube scans were excluded. Then, images of each patient were extracted and imported into the FIJI software (Rasband, W.S., ImageJ, U.S. National Institutes of Health, Bethesda, Maryland, USA, https://imagej.nih.gov/ij)^37^.

All of the imported images were processed in accordance with the comparable studies described previously^36,38,39^. After applying the mean threshold to 8-bit images, MNV complexes were extracted manually from each image using the “polygon selection” tool. Then, for further analysis, each image was binarized and skeletonized.

Vascular dispersion was computed using the “Directionality” feature of ImageJ, and fractal dimension (FD) was computed utilizing the fractal box counter of ImageJ software on binary skeleton images^1,39^.

Binarized and skeletonized images were imported into AngioTool software version 0.6 (National Institutes of Health®, Bethesda, Maryland, United States) and explant area, vessels area, vessels percentage area, total number of junctions, junctions density, total vessels length, average vessels length, total number of end points, and mean lacunarity were calculated^15,40^.

We considered mean, standard deviation, minimum, maximum, frequency, and percentage while reporting our results. Using the generalized estimating equation, we determined differences between groups, taking into account the possibility of a correlation between the measurements of both eyes of some subjects in the current study. We adjusted the p-value using the Sidak method to account for the impact of multiple comparisons on type I error. The parameters from OCTA imaging were analysed with adjustment for age and sex. All statistical analysis performed by SPSS (IBM Corp. Released 2017. IBM SPSS Statistics for Windows, Version 25.0. Armonk, NY: IBM Corp). P-values less than 0.05 were regarded as statistically significant.

## Data Availability

All data during this study are included in this article and can be directed to the corresponding author.

## References

[1] Serra R, Coscas F, Cabral D, Pinna A, Coscas G (2022). Polypoidal choroidal neovascularization versus type 1 choroidal neovascularization in age-related macular degeneration: A fractal analysis study. Retina.

[2] Cozzupoli GM (2022). InCASEOf scoring system for distinction between pachychoroid-associated macular neovascularization and neovascular age-related macular degeneration in patients older than 50 years. Sci. Rep..

[3] Sartini F, Figus M, Casini G, Nardi M, Posarelli C (2020). Pachychoroid neovasculopathy: A type-1 choroidal neovascularization belonging to the pachychoroid spectrum-pathogenesis, imaging and available treatment options. Int. Ophthalmol..

[4] Cho SC, Ryoo NK, Ahn J, Woo SJ, Park KH (2020). Association of irregular pigment epithelial detachment in central serous chorioretinopathy with genetic variants implicated in age-related macular degeneration. Sci. Rep..

[5] Miyake M (2015). Pachychoroid neovasculopathy and age-related macular degeneration. Sci. Rep..

[6] Pang CE, Freund KB (2015). Pachychoroid neovasculopathy. Retina.

[7] Sagar P, Sodhi PS, Roy S, Takkar B, Azad SV (2021). Pachychoroid neovasculopathy: A comparative review on pathology, clinical features, and therapy. Eur. J. Ophthalmol..

[8] Borrelli E (2020). Rate of misdiagnosis and clinical usefulness of the correct diagnosis in exudative neovascular maculopathy secondary to AMD versus pachychoroid disease. Sci. Rep..

[9] Viggiano P (2022). Topographical analysis of the choriocapillaris reperfusion after loading anti-VEGF therapy in neovascular AMD. Transl. Vis. Sci. Technol..

[10] Borrelli E (2018). Topographic analysis of the choriocapillaris in intermediate age-related macular degeneration. Am. J. Ophthalmol..

[11] Biesemeier A, Taubitz T, Julien S, Yoeruek E, Schraermeyer U (2014). Choriocapillaris breakdown precedes retinal degeneration in age-related macular degeneration. Neurobiol. Aging.

[12] Borrelli E, Sarraf D, Freund KB, Sadda SR (2018). OCT angiography and evaluation of the choroid and choroidal vascular disorders. Prog. Retin. Eye Res..

[13] Moreira-Neto CA, Moult EM, Fujimoto JG, Waheed NK, Ferrara D (2018). Choriocapillaris loss in advanced age-related macular degeneration. J. Ophthalmol..

[14] Moult EM (2020). Spatial distribution of choriocapillaris impairment in eyes with choroidal neovascularization secondary to age-related macular degeneration: A quantitative OCT angiography study. Retina.

[15] Choi M, Kim SW, Yun C, Oh J (2020). OCT angiography features of neovascularization as predictive factors for frequent recurrence in age-related macular degeneration. Am. J. Ophthalmol..

[16] Al-Sheikh M, Iafe NA, Phasukkijwatana N, Sadda SR, Sarraf D (2018). Biomarkers of neovascular activity in age-related macular degeneration using optical coherence tomography angiography. Retina.

[17] von der Emde L (2020). Assessment of exudative activity of choroidal neovascularization in age-related macular degeneration by OCT angiography. Ophthalmologica.

[18] Faatz H (2019). Optical coherence tomography angiography of types 1 and 2 choroidal neovascularization in age-related macular degeneration during anti-VEGF therapy: Evaluation of a new quantitative method. Eye (Lond.).

[19] Bae K, Kim HJ, Shin YK, Kang SW (2019). Predictors of neovascular activity during neovascular age-related macular degeneration treatment based on optical coherence tomography angiography. Sci. Rep..

[20] Farvardin M (2022). Pachychoroid neovasculopathy can mimic wet type age-related macular degeneration. Int. J. Retina Vitreous.

[21] Hage R (2015). Flat irregular retinal pigment epithelium detachments in chronic central serous chorioretinopathy and choroidal neovascularization. Am. J. Ophthalmol..

[22] Bousquet E (2018). Optical coherence tomography angiography of flat irregular pigment epithelium detachment in chronic central serous chorioretinopathy. Retina.

[23] Liu T, Lin W, Zhou S, Meng X (2021). Optical coherence tomography angiography of flat irregular pigment epithelial detachments in central serous chorioretinopathy. Br. J. Ophthalmol..

[24] Azzolini C (2021). The morphology of choroidal neovascularization in chronic central serous chorioretinopathy presenting with flat, irregular pigment epithelium detachment. Int. Ophthalmol..

[25] Pichi F, Morara M, Veronese C, Ciardella AP (2018). The overlapping spectrum of flat irregular pigment epithelial detachment investigated by optical coherence tomography angiography. Int. Ophthalmol..

[26] Guo J, Tang W, Liu W, Chang Q, Xu G (2021). Clinical features of flat irregular pigment epithelial detachment associated with choroidal neovascularization in chronic central serous chorioretinopathy. Retina.

[27] Dansingani KK, Balaratnasingam C, Klufas MA, Sarraf D, Freund KB (2015). Optical coherence tomography angiography of shallow irregular pigment epithelial detachments in pachychoroid spectrum disease. Am. J. Ophthalmol..

[28] Azuma K (2019). The association of choroidal structure and its response to anti-VEGF treatment with the short-time outcome in pachychoroid neovasculopathy. PLoS One.

[29] Arf S, Sayman-Muslubas I, Hocaoglu M, Ersoz MG, Karacorlu M (2020). Features of neovascularization in pachychoroid neovasculopathy compared with type 1 neovascular age-related macular degeneration on optical coherence tomography angiography. Jpn. J. Ophthalmol..

[30] Lyu X, Jajal P, Tahir MZ, Zhang S (2022). Fractal dimension of retinal vasculature as an image quality metric for automated fundus image analysis systems. Sci. Rep..

[31] Huang CH (2019). Characterizing branching vascular network morphology in polypoidal choroidal vasculopathy by optical coherence tomography angiography. Sci. Rep..

[32] Serra R (2021). Quantitative optical coherence tomography angiography features of inactive macular neovascularization in age-related macular degeneration. Retina.

[33] Altinisik M, Kurt E, Sonmezer P, Kayikcioglu O, Ilker SS (2022). A comparative study of type 1 neovascularization: Neovascular age-related macular degeneration versus pachychoroid neovasculopathy. Eur. J. Ophthalmol..

[34] Faghihi H (2021). Choroidal features in flat irregular pigment epithelial detachment associated with Chronic central serous chorioretinopathy: Avascular versus vascularized. PLoS One.

[35] Cheung CMG (2021). Polypoidal choroidal vasculopathy: Consensus nomenclature and non-indocyanine green angiograph diagnostic criteria from the asia-pacific ocular imaging society PCV workgroup. Ophthalmology.

[36] Parodi MB, Arrigo A, Bandello F (2020). Optical coherence tomography angiography quantitative assessment of macular neovascularization in best vitelliform macular dystrophy. Invest. Ophthalmol. Vis. Sci..

[37] Schindelin J (2012). Fiji: An open-source platform for biological-image analysis. Nat. Methods.

[38] Arrigo A (2018). Advanced optical coherence tomography angiography analysis of age-related macular degeneration complicated by onset of unilateral choroidal neovascularization. Am. J. Ophthalmol..

[39] Arrigo A (2020). Optical coherence tomography angiography can categorize different subgroups of choroidal neovascularization secondary to age-related macular degeneration. Retina.

[40] Nakano Y (2019). Vascular maturity of type 1 and type 2 choroidal neovascularization evaluated by optical coherence tomography angiography. PLoS One.

